# Identification of glycogene-type and validation of ST3GAL6 as a biomarker predicts clinical outcome and cancer cell invasion in urinary bladder cancer

**DOI:** 10.7150/thno.48711

**Published:** 2020-08-08

**Authors:** Sumiya Dalangood, Zhen Zhu, Zhihui Ma, Jiaxuan Li, Qinghe Zeng, Yilin Yan, Bing Shen, Jun Yan, Ruimin Huang

**Affiliations:** 1 MOE Key Laboratory of Model Animals for Disease Study, Model Animal Research Center of Nanjing University, Nanjing 210061, China.; 2 State Key Laboratory of Drug Research, Shanghai Institute of Materia Medica, Chinese Academy of Sciences, Shanghai 201203, China.; 3 University of Chinese Academy of Sciences, Beijing 100049, China.; 4 Department of Urology, Shanghai General Hospital, Shanghai Jiaotong University School of Medicine, Shanghai 200080, China.; 5 Department of Laboratory Animal Science, Fudan University, Shanghai 200032, China.; 6 Model Animal Research Center of Nanjing University, Nanjing 210061, China.

**Keywords:** glycogene, ST3GAL6, urinary bladder cancer, clinical outcome, invasion

## Abstract

**Background:** Urinary bladder cancer (UBC) is one of the most common causes of morbidity and mortality worldwide characterized by a high risk of invasion and metastasis; however, the molecular classification biomarkers and underlying molecular mechanisms for UBC patient stratification on clinical outcome need to be investigated.

**Methods:** A systematic transcriptomic analysis of 185 glycogenes in the public UBC datasets with survival information and clinicopathological parameters were performed using unsupervised hierarchical clustering. The gene signature for glycogene-type classification was identified using Limma package in R language, and correlated to 8 known molecular features by Gene Set Variation Analysis (GSVA). The clinical relevance and function of a glycogene was characterized by immunohistochemistry in UBC patient samples, and quantitative RT-PCR, Western blotting, promoter activity, MAL II blotting, immunofluorescence staining, wound healing, and transwell assays in UBC cells.

**Results:** A 14-glycogene signature for glycogene-type classification was identified. Among them, ST3GAL6, a glycotransferase to transfer sialic acid to 3'-hydroxyl group of a galactose residue, showed a significant negative association with the subtype with luminal feature in UBC patients (n=2,130 in total). Increased ST3GAL6 was positively correlated to tumor stage, grade, and survival in UBCs from public datasets or our cohort (n=52). Transcription factor GATA3, a luminal-specific marker for UBC, was further identified as a direct upstream regulator of ST3GAL6 to negatively regulate its transactivation. ST3GAL6 depletion decreased MAL II level, cell invasion and migration in 5637 and J82 UBC cells. ST3GAL6 could reverse the effects of GATA3 on global sialylation and cell invasion in SW780 cells.

**Conclusions:** Herein, we successfully identified a novel 14-gene signature for glycogene-type classification of UBC patients. ST3GAL6 gene, from this signature, was demonstrated as a potential biomarker for poor outcomes and cell invasion in UBCs.

## Introduction

Urinary bladder cancer (UBC) is one of the most common causes of morbidity and mortality worldwide characterized by a high risk of invasion, metastasis and recurrence 1. These tumors are staged using the Tumor-Node-Metastasis (TNM) system, as non-muscle-invasive bladder cancer (NMIBC; Tis, Ta, and T1) and muscle-invasive bladder cancer (MIBC; T2, T3, and T4) according to the extent of invasions. Ta tumors are restricted to the urothelium; T1 tumors have invaded the lamina propria; and T2, T3, and T4 tumors have invaded the superficial muscle, perivesical fat, and surrounding organs, respectively 2. UBCs could be graded according to cellular characteristics as papillary urothelial neoplasm of low malignant potential (PUNLMP), low grade and high grade papillary urothelial carcinoma in the 2004 WHO/ISUP criteria 3. Current prognostication and clinical management are highly based on above basic histopathologic evaluation.

The intratumoral and intertumoral heterogeneity at the genomic, transcriptional and cellular levels contribute to the capricious outcomes of UBC patients 4. Even the pathologically similar, the intrinsic molecular and genetic events were quite different; thus, a number of groups used gene expression patterns to reveal the molecular subtypes which traverse stage and grade classification 5-10. For MIBCs, the luminal-like subtype was characterized by the expression of transcription factors and markers for differentiation (GATA3, FoxA1 and KRT20) 11, 12; whereas, the basal-like subtype was enriched with cancer stem cell, mesenchymal-like markers (KRT14, KRT5, CD44, and Snails) and squamous differentiation markers (TGM1 and PI3) 9. Such molecular classification helps to a precise prediction of UBC outcomes and therapeutic interventions. For example, the luminal subtype with papillary feature had the longest 5-year survival; the luminal subtype with EMT feature and basal subtype with squamous differentiation feature had second survival performance; the subtype with neuroendocrine feature had the worst clinical outcomes 9. However, complexity of the bioinformatics, diversity of the subtypes, even labor and cost for the microarray analyses hurdle the clinical practice of molecular taxonomy stratification in UBCs 7, 13, 14. A novel clinical- and molecular-related biomarker for UBC outcomes is urgently needed.

Glycosylation represents a post-translational modification that involves the enzymatic linkage of monosaccharides or whole oligosaccharides (glycans) to specific amino acids within proteins 15, 16. The glycosylation-related genes, including glycosyltransferases, glycosidases, and nucleotide sugar synthesis and transporter genes, are named as "glycogenes" and equivalent to 1% of human genome 17, 18. As a potential cancer hallmark, aberrant glycosylation plays an important role in tumor initiation, progression and metastasis 19. The *de novo* expression of certain antigens such as sialyl Lewis A (sLe^A^) and sialyl Lewis X (sLe^X^) are frequently detected in many cancers, and associated with poor prognosis 20. For instance, CA19-9 is a sLe^A^-type glycan epitope, serves as a routine serum biomarker for pancreatic and gastric cancers 21. Dysregulated glycosyltransferases, such as sialyltransferase 22, at the level of transcription and/or post-transcription were associated with cancer cell invasion 23, migration and chemoresistance 24. It is thus intriguing and important to identify the glycogene-type and/or glycosyltransferases as the biomarkers for molecular stratification in UBCs.

Herein, we divided UBC patients into four glycogene-type based subtypes with different clinical outcomes, using the transcriptomic data and clinical information from multiple public UBC datasets. A 14-glycogene signature was then successfully identified to predict the outcomes of UBC patients. After the comparison with the known subtype markers, ST3GAL6 (a sialyltransferase) was selected because it was downregulated in the luminal subtype and upregulated in the non-luminal subtypes, as a promising biomarker for poor prognosis of UBCs. We further demonstrated that ST3GAL6 was negatively regulated by a luminal-specific transcription factor GATA3, and knockdown its expression suppressed the invasion capability of UBC cells.

## Methods

### Unsupervised hierarchical clustering and assembly of the TCGA and GEO datasets

Gene expression and clinical data from the UBC cohort in TCGA database (TCGA-BLCA) were downloaded from Genomic Data Commons Data Portal (https://portal.gdc.cancer.gov/). The FPKM expression of each gene was applied with Log(1+FPKM) and normalized with Z-score (mean-centered). Gene expression and clinical data from MSK (JCO 2013) dataset were collected from Cbioportal (https://www.cbioportal.org/) 25-27. Medium expression of genes from this dataset was applied in the following analysis. Other public microarray data as well as the corresponding clinical data were obtained from the Gene Expression Omnibus (GEO) database (http://www.ncbi.nlm.nih.gov/geo). Gene expression in GEO were normalized with Z-score (mean-centered). In the case one gene with the multiple probes, the averaged expression was used. 185 glycogenes were obtained from the glycogene database (GGDB, https://acgg.asia/ggdb2/) and the previous reports 18, 28, 29.

Unsupervised hierarchical clustering of TCGA and GEO datasets were indicated in Morpheus (https://software.broadinstitute.org/morpheus) by Average Linkage method with One minus Pearson correlation. Different expressed genes in TCGA-BLCA dataset were analyzed using Limma, an R package using the linear models to assess the differential expression, based on the Log(1+FPKM). Glycogenes exhibited remarkable differential expression between B1 subcluster and other subclusters (A1, A2 and B2; Log_2_ Fold Change > 1.5, p < 0.05, and FDR < 0.01) and the confirmation study was performed in other three independent datasets (MSK (JCO 2013), GSE13507, and GSE32894).

To investigate the relationship between glycogene-types and molecular features, data from 12 independent datasets (TCGA-BLCA, MSK (JCO 2013), GSE13507, GSE32894, GSE48276, GSE128702, GSE48075, GSE87304, GSE32584, GSE31684, GSE128192 and GSE3167; n=2,130 in total) were analyzed using Gene Set Variation Analysis (GSVA) 30. Gene sets for luminal markers (*CYP2J2*, *ERBB2*, *ERBB3*, *FGFR3*, *FOXA1*, *GATA3*, *GPX2*, *KRT18*, *KRT19*, *KRT20*, *KRT7*, *KRT8*, *PPARG*, *XBP1*, *UPK1A*, and *UPK2*), basal markers (*CD44*, *CDH3*, *KRT1*, *KRT14*, *KRT16*, *KRT6B*, and *KRT6C*), squamous-differentiation markers (*DSC1*, *DSC2*, *DSC3*, *DSG1*, *DSG2*, *DSG3*, *S100A7*, and *S100A8*), epithelial-mesenchymal markers (*ZEB1*, *ZEB2*, *VIM*, *SNAIL*, *TWIST1*, *FOXC2*, and *CDH2*), Claudin-low markers (*CLDN3*, *CLDN7*, *CLDN4*, *CDH1*, *VIM*, *SNAI2*, *TWIST1*, *ZEB1*, and *ZEB2*), p53-like markers (*ACTG2*, *CNN1*, *MYH11*, *MFAP4*, *PGM5*, *FLNC*, *ACTC1*, *DES*, and *PCP4*), neuroendocrine markers (*CHGA*, *CHGB*, *SCG2*, *ENO2*, *SYP*, and *NCAM1*), and cancer-stem cell markers (*CD44*, *KRT5*, *RPSA*, and *ALDH1A1*) were adapted from the previous reports 9, 31.

### Patient sample collection and immunohistochemistry (IHC)

Fifty-two patients who initially diagnosed as primary UBC were enrolled and samples were collected after surgery in Shanghai General Hospital (Shanghai, China). This study was approved by the Institutional Review Board of Shanghai General Hospital, with the written informed consent from the corresponding patients. The formalin-fixed paraffin embedded tissues were cut and 5 μm-thick sections were collected on super-frost, positively charged glass slides. IHC was carried out as described previously 32. Briefly, after antigen retrieval by autoclave in citrate buffer at pH 6, sections were incubated with primary anti-ST3GAL6 antibody (1/100, ab106527; Abcam, USA) overnight at 4 °C, followed by the incubation with the horseradish peroxidase (HRP)-conjugated antibody. The slides were visualized by DAB visualization kit (DAB-0031; Maixin_Bio, China), counterstained with hematoxylin. Images were acquired by a slide scanner (NanoZoomer 2.0-HT; HAMMATSU, Japan) and analyzed by NDP Serve slide distribution and management software (HAMMATSU). IHC slides were evaluated by two pathologists independently. The highest intensity was initially scored as 5 and the lowest was scored as 1; and the final score of the ST3GAL6 staining was the multiplication of the intensity value and the positive ratio value 33.

### Cell culture and reagents

UBC cell lines, including RT4, RT112, 5637, J82, T24, SW780, UMUC-3, UMUC-14 and HT1376 were obtained from Cell Bank of Type Culture Collection, Chinese Academy of Sciences (Shanghai, China). UMUC-3, UMUC-14 and HT1376 cells were maintained with EMEM medium and other cells were cultured with RPMI1640 medium at 37 °C in a humidified atmosphere of 5% CO_2_. Both medium contained 10% FBS and penicillin/streptomycin (Invitrogen, USA). Knockdown experiments were conducted using siRNAs targeting ST3GAL6 or GATA3 or negative control (siNC) with Lipofectamine RNAiMAX reagent (Invitrogen), according to manufacturer's instructions. Knockdown experiment using shRNAs were performed as previously reported 32. shRNA plasmids targeting ST3GAL6 were generated using the lentiviral pLKO.1 backbone with puromycin resistance. The sequences for each siRNA and shRNA were listed in Table S1.

### RNA extraction and quantitative Reverse Transcription PCR (qRT-PCR) analyses

After total RNAs were extracted from UBC cells by Trizol reagent (Invitrogen), 2 μg of total RNA was reverse transcribed to cDNA by the QuantiTect Reverse Transcription Kit (QIAGEN, USA). RT-PCR analyses were performed by AceQ Universal SYBR qPCR Master Mix (Q511-02; Vazyme, China) on LightCycler 96 detection system (BioRad, USA). Primers for qRT-PCR were listed in Table S1. mRNA expression levels were determined by the 2^-ΔΔct^ method and relative mRNA levels of interested genes were normalized to β-actin mRNA level. Each experiment was repeated three times.

### Western blotting

Total protein was extracted using RIPA lysis buffer supplemented with EDTA-free Protease Inhibitor Cocktail (#4693132001; Roche, Germany), and were boiled in SDS loading buffer. 10 μg protein was separated by 10% SDS-PAGE, followed by transferring onto 0.45 μm nitrocellulose membranes (Millipore, USA). The membranes were blocked with 5% non-fat milk in PBST and incubated with primary antibodies against ST3GAL6 (1/500, ab106527; Abcam), GATA3 (1/500, #558686; BD, USA), EGFR (1/1000, #4267; Cell Signaling Technology (CST), USA), phospho-EGFR (Tyr1068) (1/1000, #3777; CST), AKT (pan) (1/1000, #4691; CST), phospho-AKT (Ser473) (1/1000, #4060; CST), p44/42 MAPK (ERK1/2) (1/1000, #4695; CST), phospho-p44/42 MAPK (ERK1/2) (Thr202/Tyr204) (1/1000, #4370; CST), STAT3 (1/1000, #9139; CST), phospho-STAT3 (Tyr705) (1/1000, #9145; CST), or GAPDH (1/1,000, sc-47724; Santa Cruz Biotechnology, USA) at 4℃ overnight. After rinse with PBST three times, the membranes were incubated with HRP-conjugated anti-rabbit or anti-mouse antibodies (1/5,000; Jackson ImmunoResearch, USA; Table S1), followed by the detection with the Super Signal West Pico PLUS Chemiluminescent Substrate (Thermo Scientific, USA).

### Lectin blotting and silver staining

Protein samples (10 μg) were separated by 10% SDS-PAGE and transferred to 0.45 μm nitrocellulose membranes (Millipore). The membranes were incubated with the biotinylated Maackia amurensis Lectin II (MAL II; 1/1,000, B-1265; Vector Laboratories, USA) overnight at 4 °C. After rinse with TBST three times, the membranes were incubated with HRP-conjugated streptavidin (1×, SA-5704; Vector Laboratories) for 1 h and detected with the Super Signal West Pico PLUS Chemiluminescent Substrate. Silver staining was carried out using Fast Silver Stain Kit (P0017S; Beyotime Biotechnology, China). Briefly, after electrophoresis and acid fixation, SDS-PAGE gels were impregnated with the Sensitizing solution, followed by washing with ultrapure ddH_2_O. Gels were placed in the Staining solution till the desired band intensity was achieved. The Stopper buffer was immediately added directly to the gel to terminate the reaction. The gels were photographed.

### Immunofluorescence staining

Cells were fixed with 4% paraformaldehyde for 30 min and permeabilized with 0.5% Triton X-100 for 20 min. After washed by PBS three times, cells were blocked with 5% BSA for 30 min and then incubated with biotinylated-MAL II (Vector Laboratories) overnight at 4 °C. Cells were incubated with FITC-Avidin (#405101; BioLegend, USA) for 1 h. The cells were washed by PBST and counterstained with 2 μg/mL Hoechst (#33342; Thermo Scientific) for 5 min. Images were obtained using a fluorescence microscope (DM6 B; Leica, Germany).

### Promoter activity assay

For promoter analysis assay, the fragment of human ST3GAL6 gene promoter containing wild-type (WT) GATA3 binding site was cloned into pGL3-Basic luciferase reporter vector (Promega, USA), and the reporter containing the mutant (MT) GATA3 binding site was then generated using Mut Express II Fast Mutagenesis Kit V2 (C214-02; Vazyme). The human GATA3 cDNA was cloned into pCDH-3×FLAG plasmid to obtain GATA3 expression construct. The primers for cloning were listed in Table S1. The pGL3-ST3GAL6-WT-Luc (0.2 μg) or pGL3-ST3GAL6-Mutant-Luc plasmid (0.2 μg) was transfected by Lipofectamine 3000 (Invitrogen) into 5637 cells in 24-well plate, along with 1 ng pRL-CMV plasmid (Promega) and 0.2 μg or 0.4 μg pCDH-3×FLAG-GATA3. After 24 h, cell lysates were collected. Luciferase activities were measured using the dual luciferase reporter assay system (RG027; Beyotime Biotechnology) by an *In Vivo* Imaging System (IVIS) Spectrum (PerkinElmer, USA). Firefly luciferase activity was normalized to renilla luciferase activity for the relative promoter activity. Experiments were performed in triplicate.

### Cell proliferation assay

Cell proliferation was assessed by the Cell Counting Kit-8 (CCK-8; Vazyme). Briefly, 2×10^3^ cells/well were plated in 96-well plates and 10 μL CCK8 solution was added into each well 2 h before detection at the indicated time points. The absorbance at 450 nm (A450) was examined by Synergy H1 hybrid multi-mode reader (BioTek, USA).

### Wound healing assay

2×10^5^ cells were seeded on a 12-well plate till confluency. A wound was made by scratching with a pipette tip, followed by wash with PBS. The wound closure was captured by a microscope (ECLIPSE Ti; Nikon, Japan) at different time points (0 h and 24 h).

### Transwell assay

Invasion assay was performed using Corning BioCoat (Matrigel matrix) Tumor Invasion Systems (FluoroBlok PET Membrane, 8.0 μm; 24-Multiwell) (#354165; Corning, USA). In brief, cells were serum-starved overnight, harvested and re-suspended in the migration medium, a suspension of 2×10^5^ cells in 100 μL of serum-free medium was seeded on top of transwell. Complete medium with serum was placed in the lower compartment of the chamber as a chemoattractant. After 24 h, transwells were fixed by 4% paraformaldehyde and stained by 0.2% crystal violet. The cells on the upper side were removed by Q tips. The invasive capacity was evaluated by counting the invading cells under a microscope (Nikon). Three random fields of view were analyzed for each chamber.

### Statistical analysis

Fisher exact test, χ^2^ test and unpaired *t* test (two-tailed) were used among clusters and variables. Pearson correlation was used to determine the correlation between gene and gene or the correlation between gene and gene sets. Kaplan-Meier survival curves with log-rank test were applied to analyze overall survival (OS), disease free survival (DFS), and cancer-specific survival. Univariate and multivariate models were computed using Cox proportional hazards regression by “survival” and “survminer” in R package, respectively. Statistical analysis was performed by GraphPad Prisms 8.0 software (GraphPad Software Inc., USA) or Bioconductor/R. Data were presented as means ± standard deviation (SD) from at least three independent experiments. In all analysis, *p* values less than 0.05 were considered statistically significant.

## Results

### Molecular classification based on the expression pattern of glycogenes correlated to prognosis of UBC patients

**Figure 1:**
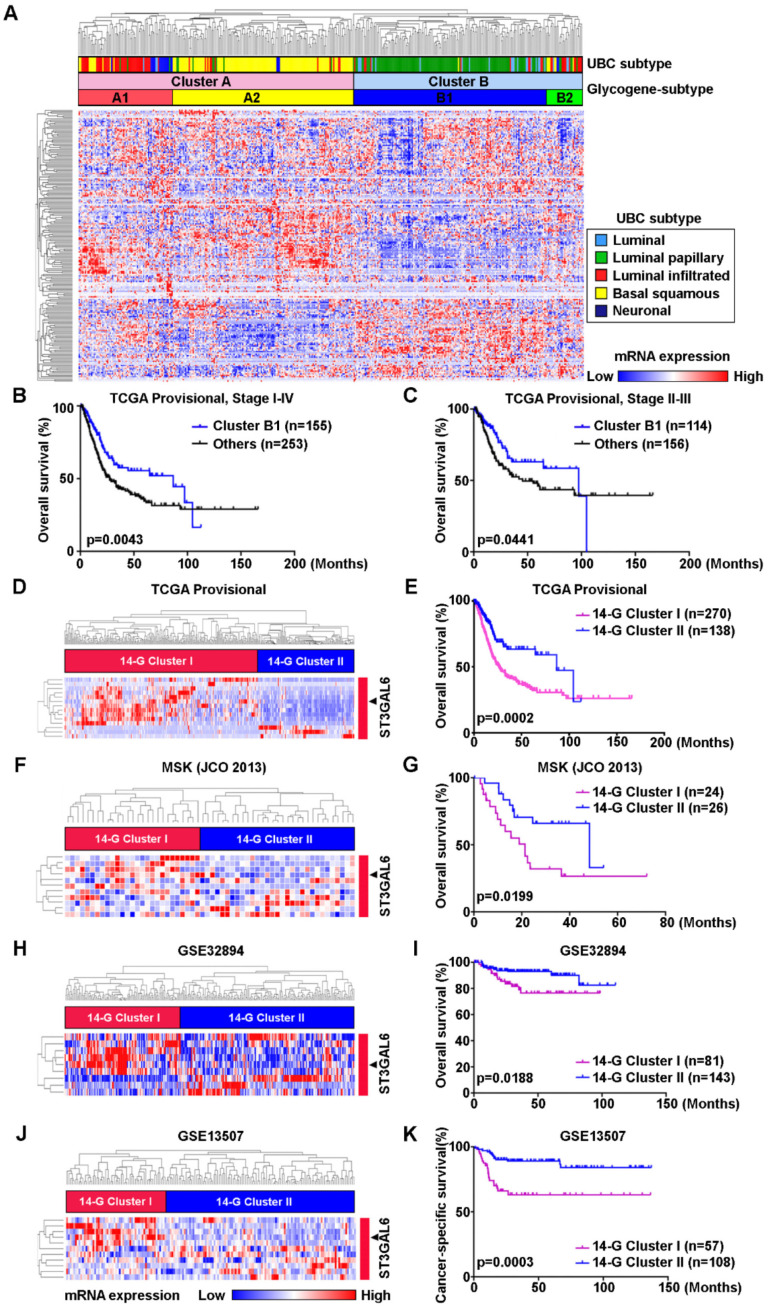
** Identification of glycogene-based subtypes in UBCs. A**, Unsupervised clustering analysis using 185 glycogenes divided the UBC patients from TCGA provisional dataset (n=408) into two clusters (Cluster A and B) or four subclusters (A1, A2, B1, and B2). Five molecular subtypes were also indicated. **B**-**C**, Kaplan-Meier plot of overall survival of UBC patients (Stage I-IV, **B**; Stage II-III, **C**) in TCGA provisional dataset, comparing that in subcluster B1 with other three subclusters (A1, A2 and B2). **D**, **F**, **H**, and **J**, A 14-glycogene signature, which was identified to be significantly dysregulated in subcluster B1 comparing with subclusters A1, A2 and B2 in TCGA provisional dataset (Figure S2A), allowed the glycogene-based classification (14-G Cluster I and II) in UBC patients from TCGA provisional (**D**), MSK (JCO 2013, **F**), GSE32894 (**H**) and GSE13507 (**J**) datasets. **E**, **G**, **I**, and **K**, Kaplan-Meier plot of overall survival or cancer-specific survival of UBC patients from TCGA provisional (**E**), MSK (JCO 2013, **G**), GSE32894 (**I**) and GSE13507 (**K**) datasets, by glycogene-type classification as 14-G Cluster I and II.

To investigate whether the abnormal glycogene expression defines a molecular subtype of UBC patients, we designed the study with the details shown in Figure S1. An unsupervised hierarchical clustering was carried out using 185 unique glycogenes in UBC patients from TCGA-BLCA (TCGA provisional dataset; n=408). The result demonstrated that UBC patients can be categorized into two clusters (A and B) or four subclusters (A1, A2, B1 and B2; Figure 1A). UBC patients in this dataset have been well defined as five molecular subtypes, including Luminal papillary (~35%), Luminal infiltrated (~19%), Luminal (~6%), Basal squamous (~35%), and Neuronal (~5%), based on the mRNA expression pattern combining BayesNMF with a consensus hierarchical clustering approach 9. The strong correlation between our four- subcluster and above five-subtype classification was observed (Figure 1A). The association between subcluster B1 and Luminal papillary subtype, as well as the association between subcluster A2 and Basal squamous subtypes were especially notable. In addition, the patients in subcluster B1 were negatively associated with tumor grade (*p* < 0.0001) and tumor stage (*p* < 0.0001; Table S2), and showed significantly better prognosis than other subclusters for all stages (I-IV) (*p* = 0.0043; Figure 1B) or for stages II-III (*p* = 0.0441; Figure 1C). It is indicated that the novel glycogene expression-based profiling (glycogene-type) may also be suitable for UBC classification with clinical outcomes.

To simplify the gene signature to differentiate the subcluster B1 from other three subclusters, 14 glycogenes (*B4GALNT1, B4GALNT2, CHSY3, FUT7, GALNT17, GGTA1P, GLT1D1, GLT8D2, GXYLT2, ST3GAL6, ST6GALNAC5, UGT2B4, UGT2B15, and UGT2B28*) were identified to be significantly dysregulated in subcluster B1, comparing with the other three subclusters, in the TCGA provisional dataset (*p* < 0.01; Table S3 and Figure S2A). The 14-glycogene signature enabled the division of the UBC patients by two clusters (14-G Cluster I and II; Figure 1D) with significant OS differences (*p* = 0.0002, Figure 1E; and HR (95% CI) = 1.982(1.380-2.849), *p* = 0.0002, by univariate analysis, Table S4). Another 3 independent datasets, including MSK (JCO 2013; n=50), GSE32894 (n=224) and GSE13507 (n=165), were enrolled to validate this 14-glycogene signature (Figure 1F, 1H, 1J, and Figure S2B-E). Remarkable differences of OS or cancer-specific survival also existed between 14-G Cluster I and 14-G Cluster II in these three datasets (*p* < 0.05, Figure 1G, 1I, and 1K; HR (95% CI) = 2.500(1.133-5.513), *p* = 0.023, by univariate analysis for OS in GSE32894, Table S5; HR (95% CI) = 3.447(1.699-6.993), *p* = 0.001, by univariate analysis for cancer-specific survival in GSE13507, Table S6). These data indicate the 14-glycogene signature may define the new subtypes in UBC patients.

### ST3 β-galactoside alpha-2,3-sialyltransferase 6 (ST3GAL6) was negatively correlated to the subtype with luminal feature in UBC patients

**Figure 2:**
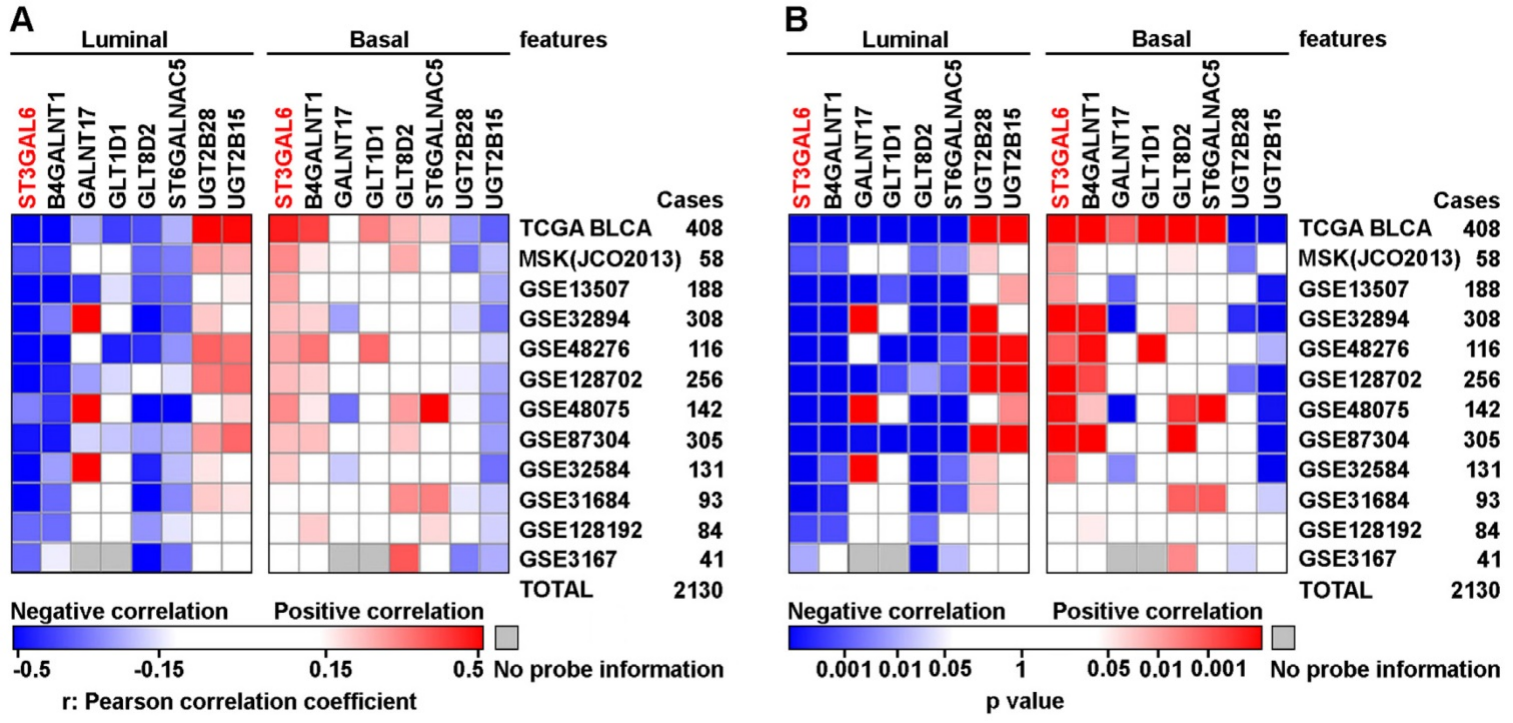
** The correlations between mRNA expressional levels of 14-glycogene signature and molecular features in UBC patients.** Pearson correlation analyses (r value, **A**) with the statistical significance of correlation (p value, **B**) were performed between the expression levels of 8 glycogenes from the 14-gene signature with the complete probe information and 2 molecular features (Luminal and Basal) in 12 independent cohorts (n=2,130 in total). Colored-blocks in the heatmaps represented the glycogenes with upregulation (in red) or downregulation (in blue), respectively.

The relevance between 14-glycogene signature and molecular features, such as luminal, basal, squamous-differentiation, epithelial-mesenchymal, cancer-stem cell, Claudin-low, p53-like, and neuroendocrine, was analyzed in 12 independent datasets (n=2,130 in total) using GSVA for the feature scores respectively. Because only 8 from the 14 glycogenes with complete probe information in all datasets, correlations between mRNA expression levels of these 8 genes and above 8 feature scores were then determined by Pearson correlation test. Notably, ST3GAL6 gene expression showed a negative association with luminal feature, along with positive associations with other features (including basal feature) in the majority of 12 datasets (Figure 2 and Figure S3). An individual glycogene, ST3GAL6, as a potential classification candidate for UBC patients is suggested.

### Increased expression of ST3GAL6 was associated with poor prognosis in UBC patients

**Figure 3:**
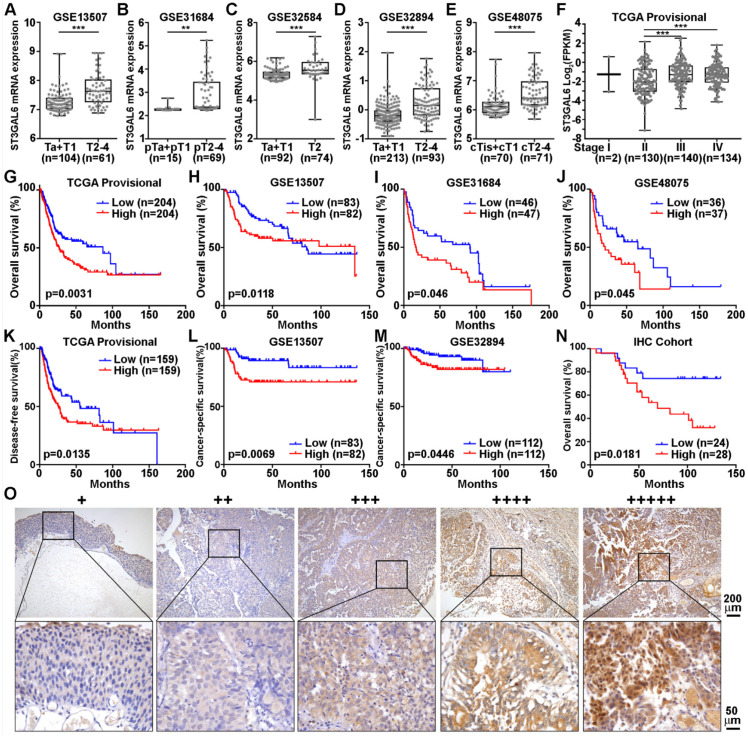
** The association of ST3GAL6 expression and clinical features. A-F**, The correlations between ST3GAL6 mRNA levels and tumor stage of UBC patients from GES13507 (**A**), GES31684 (**B**), GES32584 (**C**), GES32894 (**D**), GES48075 (**E**), and TCGA provisional (**F**) datasets. **G-J**, Kaplan-Meier plot of overall survival of UBC patients in TCGA provisional (**G**), GES13507 (**H**), GSE31684 (**I**) and GSE48075 (**J**) datasets, stratified by ST3GAL6 expression. **K-M**, Kaplan-Meier plot of disease-free survival or cancer-specific survival of UBC patients in TCGA provisional (**K**), GES13507 (**L**), and GSE32894 (**I**) dataset, stratified by ST3GAL6 expression. **N**, Kaplan-Meier plot of cumulative overall survival of UBC patients in our IHC cohort, using the mean value of ST3GAL6 IHC staining scores as the cutoff point. **O**, IHC staining of ST3GAL6 in UBC patients. The representative images for different staining intensities were shown. ***, p < 0.001; **, p < 0.01.

To further validate whether ST3GAL6 was involved in UBC development, 6 datasets with tumor stage information (n=1,268 in total) and 5 from these 6 datasets with tumor grade information (n=1,124 in total) were analyzed. ST3GAL6 mRNA expression was significantly elevated in high stages (T2-4), compared with that in low stages (Ta+T1 or Tis+T1) (*p* < 0.01; Figure 3A-E). Even in the MIBC subgroup within the TCGA provisional dataset, we observed that ST3GAL6 mRNA expression was much higher in Stage III or Stage IV than that in Stage II (*p* < 0.001; Figure 3F). Overexpressed ST3GAL6 in high grade UBCs was also shown (*p* < 0.05; Figure S4A-E). In the TCGA provisional dataset, up-regulation of ST3GAL6 was observed in UBC patients with lymph node metastasis (≥N1; n=128) or with recurrence (Recurred; n=141), comparing to those without lymph node metastasis (N0; n=235, *p* < 0.05; Figure S4F) or without recurrence (DiseaseFree; n=178, *p* < 0.01; Figure S4G), respectively.

We also tested whether ST3GAL6 expression level was associated with patients' outcomes. The patients were divided into two groups, with low or high expression of ST3GAL6, using median values as the cutoff points. The Kaplan-Meier survival analysis showed that the ST3GAL6 expression level was inversely associated with OS in all 4 datasets (n=739 in total, *p* < 0.05; Figure 3G-J) and disease-free/cancer-specific survival in all 3 datasets (n=707 in total, *p* < 0.05; Figure 3K-M), respectively.

Furthermore, we collected 52 UBC specimens (IHC cohort) to validate the results from public datasets. IHC staining showed that ST3GAL6 protein was mainly located in the cytosol of UBC cells (Figure 3O). High ST3GAL6 protein level was also significantly associated with tumor grade (*p* = 0.029; Table S7) and poor OS (*p* = 0.0181, Figure 3N; HR (95% CI) = 2.951(1.149-7.579), *p* = 0.025, by univariate analysis for OS, Table S8).

In summary, increased expression of ST3GAL6 gene was demonstrated to be correlated to poor prognosis in UBC patients from both public datasets and our own cohort.

### Negative correlation of expression levels between ST3GAL6 and GATA3 in UBC patients and cells

**Figure 4:**
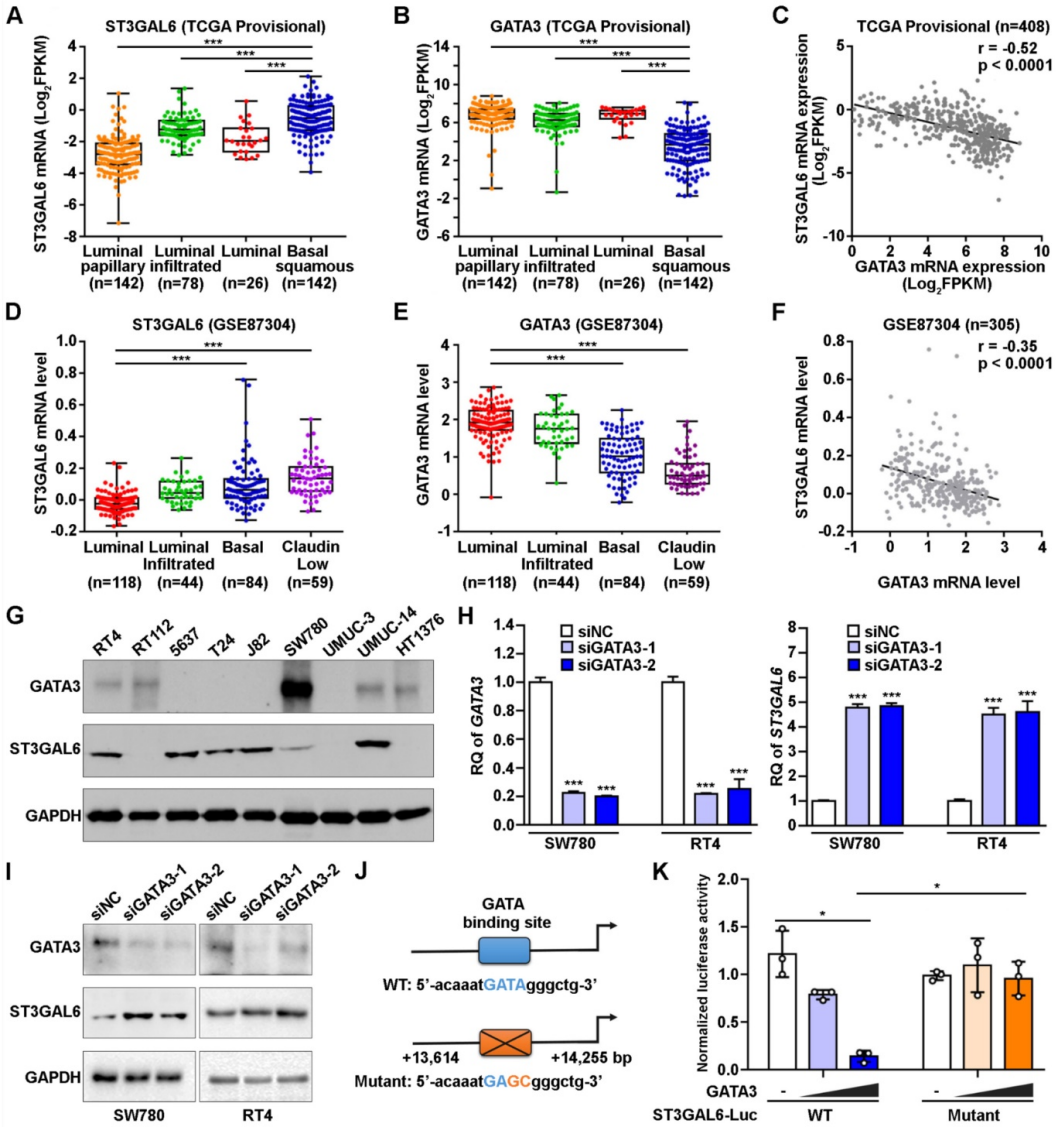
** The correlation of GATA3 and ST3GAL6 expression in UBC samples and cells. A-C**, The mRNA levels of ST3GAL6 (**A**) and GATA3 (**B**) in four subtypes, along with their Pearson correlation (**C**) in UBC patients from TCGA provisional dataset. **D-F**, The mRNA levels of ST3GAL6 (**D**) and GATA3 (**E**) in four subtypes, along with their Pearson correlation (**F**) in UBC patients from GSE87304 dataset. **G**, The protein levels of ST3GAL6 and GATA3 in 9 UBC cell lines by Western blotting. **H-I**, mRNA and protein expression levels of ST3GAL6 and GATA3 in SW780 and RT4 cells transfected with siRNAs targeting GATA3 (siGATA3-1 or -2) or control siRNA (siNC), detected by qRT-PCR (**H**) and Western blotting (**I**), respectively. **J**, Sequences for ST3GAL6 luciferase reporters, ranging between 13,614 and 14,255 bp from the transcriptional start site of *ST3GAL6* (NM_001323360). Wildtype (WT, in blue) and mutant (in orange) GATA binding sites were indicated. **K**, The activities of ST3GAL6 luciferase reporters (WT and mutant) in the presence of GATA3 expression plasmids (0.2 and 0.4 µg/well) from 5637 cells in 24-well-plate, normalized by activities of co-transfected pRL-CMV. Data were presented as mean ± SD of three independent experiments; ***, p < 0.001; *, p < 0.05.

It is important to explore which upstream factors regulate the expression of ST3GAL6 gene. Since our results suggested a negative association between ST3GAL6 mRNA expression and UBC patients with luminal feature (Figure 2), we analyzed the expression levels of ST3GAL6 mRNA in TCGA provisional and GSE87304 datasets which had molecular classification information. Significantly lower ST3GAL6 expression was shown in all luminal-related subtypes (Luminal, Luminal papillary, and Luminal infiltrated; n=246 in total), compared with that in Basal squamous subtype (n=142) (TCGA provisional dataset, *p* < 0.001; Figure 4A). Similar result was observed between the Luminal subtype (n=118) and Basal subtype (n=84) (GSE87304 dataset, *p* < 0.001; Figure 4D). Thus, the luminal subtype-specific transcriptional factors whose expression level was negatively associated with ST3GAL6 mRNA level were investigated in 7 independent datasets. GATA3, which was upregulated in Luminal-related subtypes and downregulated in Basal-related subtypes (*p* < 0.001; Figure 4B and 4E) 11, was identified because of the negative correlation with ST3GAL6 expression (*r* = -0.52 for TCGA provisional, *r* = -0.35 for GSE87304, *r* = -0.56 for GSE13507, *r* = -0.52 for GSE31684, *r* = -0.60 for GSE32584, *r* = -0.55 for GSE32894, and *r* = -0.66 for GSE48075;* p* < 0.001; Figure 4C, 4F and Figure S5).

Whether GATA3 directly regulates ST3GAL6 gene expression at transcriptional level was further investigated. GATA3 and ST3GAL6 protein levels were examined in 9 UBC cell lines. We found that GATA3 was relatively higher expressed in two luminal-type UBC cell lines (RT4 and SW780), but lower in basal-type 5637 cells 11; while ST3GAL6 was expressed relatively lower in RT4 and SW780 cells, but higher in 5637 cells (Figure 4G). Knocking down GATA3 by siRNAs, upregulated ST3GAL6 mRNA and protein in RT4 and SW780 cells by quantitative RT-PCR (*p* < 0.001; Figure 4H) and Western blotting (Figure 4I), respectively. Moreover, a conserved putative GATA3 binding site was found in the intron 1 of *ST3GAL6* gene locus (Figure S6A-C), whose location was similar to the reported GATA3 target genes, such as *ITM2A* 34. ST3GAL6 luciferase reporters were generated, driven by the ST3GAL6 promoter fragment containing the wildtype (WT) or mutant GATA binding site (Figure 4J). Ectopically expressed GATA3 decreased the luciferase activity of ST3GAL6 WT reporter, but not mutant reporter, in 5637 cells (*p* < 0.05; Figure 4K) and 293T cells (*p* < 0.001; Figure S6D). These results indicated that ST3GAL6 is a direct target of transcriptional factor GATA3, which may repress ST3GAL6 expression in luminal-related subtypes of UBCs.

### ST3GAL6 depletion resulted in the decrease of global sialylation level and cell invasion

**Figure 5:**
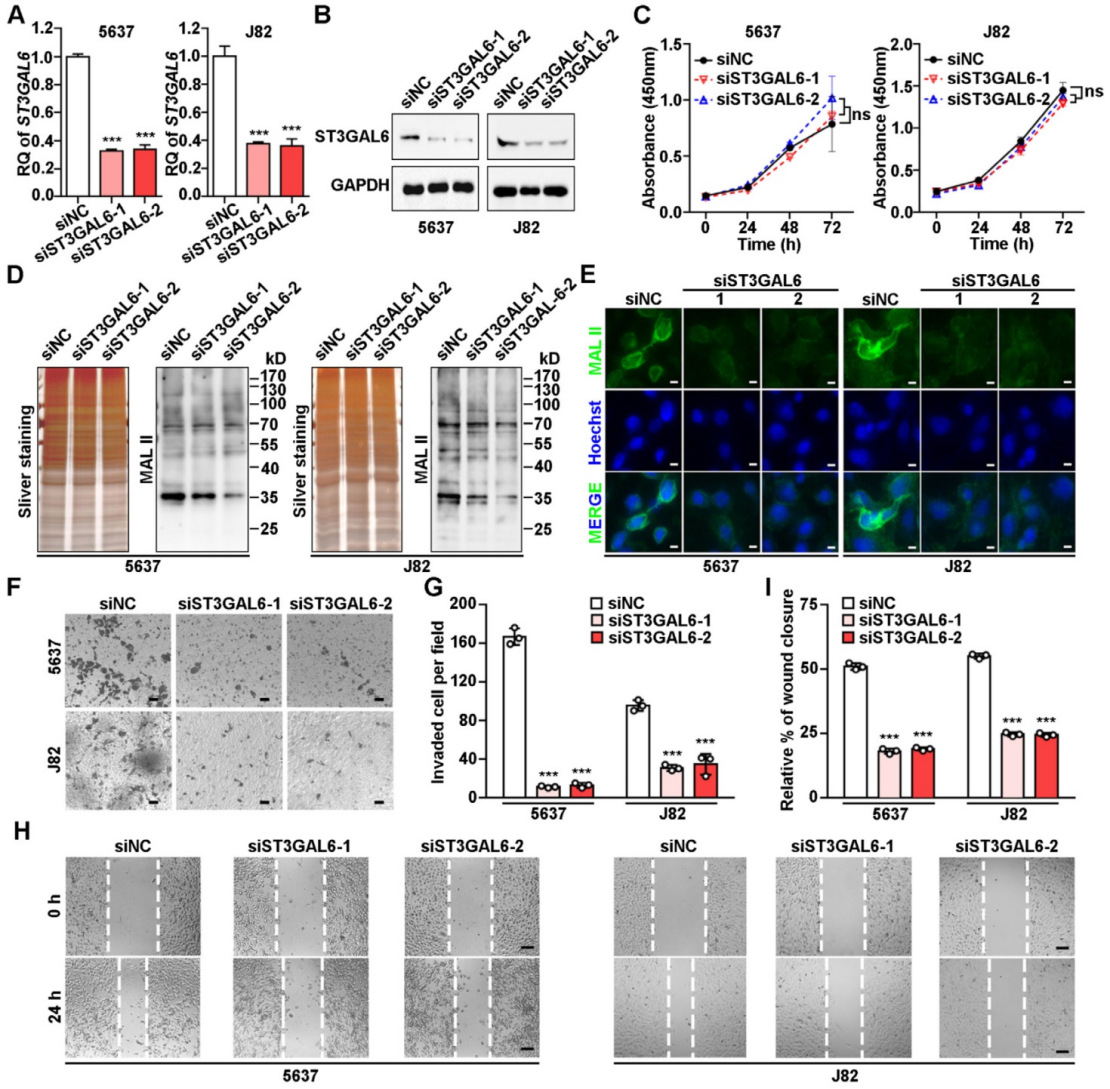
** Biological effects of ST3GAL6 depletion in UBC cells. A**-**B**, mRNA and protein expression levels of ST3GAL6 gene in 5637 and J82 cells transfected with siRNAs targeting ST3GAL6 (siST3GAL6-1 or -2) or control siRNA (siNC), detected by qRT-PCR (**A**) and Western blotting (**B**), respectively. **C**, The cell proliferation of 5637 and J82 cells with the depletion of ST3GAL6 by CCK-8 assay. **D**-**E**, Global sialylation levels in 5637 and J82 cells transfected with ST3GAL6 siRNAs, detected by MAL II blotting (**D**) and immunofluorescence staining (**E**, upper panel), respectively. The silver staining blots (**D**) were used as loading controls. Hoechst 33253 was used for the nuclei staining (**E**, middle panel). Scale bars, 10 µm. **F** and **H**, Cell invasion and migration capacities in 5637 and J82 cells transfected with ST3GAL6 siRNAs, detected by transwell invasion (**F**) and wound healing (**H**) assays, respectively. Scale bars, 50 µm. **G** and **I**, Quantifications of images for transwell invasion (**F**) and wound healing (**H**) assays. Data were presented as mean ± SD of three independent experiments; ***, p < 0.001; ns, p ≥ 0.05.

To understand the biological function of ST3GAL6 in tumor development, two UBC lines (5637 and J82) with relatively high ST3GAL6 protein levels (Figure 4G) were selected for further analyses. Knockdown of ST3GAL6 by two specific siRNAs (siST3GAL6-1 and siST3GAL6-2) was confirmed at both mRNA (Figure 5A) and protein levels (Figure 5B). However, downregulated ST3GAL6 did not have significant effect on cell proliferation (Figure 5C). Since ST3GAL6 is a member of the sialyl-transferase family that could transfer sialic acid to terminal positions on sialylated glycolipids (gangliosides) or to the N- or O-linked glycosylation, Lectin blotting and immunofluorescence staining for MAL II were performed to evaluate the transferase activity of ST3GAL6. It was demonstrated that inhibition of ST3GAL6 expression led to the decreased signals of MAL II on membranes (Figure 5D) and in cells (Figure 5E), indicating that α-2,3 sialylation was suppressed in 5637 and J82 cells. It was reported that the α2,3-sialylation levels of EGFR were significantly decreased in the ST3GAL6 knockout HeLa cells; whereas overexpression of ST3GAL6 sufficiently rescued the total α2,3-sialylation levels and α2,3-sialylation of EGFR 35. It is well known that downstream effects of EGFR signaling include regulations on three major pathways, RAS-RAF-MEK-ERK, PI3K-AKT and JAK-STAT pathway, resulting in activation of cell survival, cell proliferation, cell migration and invasion 36; thus, we examined the phosphorylation levels of ERK, AKT and STAT3 in ST3GAL6-knockdown 5637 UBC cells by two short hairpin RNAs (shRNAs) targeting different regions of ST3GAL6 mRNA (shST3GAL6-1 and shST3GAL6-2). As Figure S7A shown, the phosphorylation levels of AKT and STAT3 were decreased remarkably in ST3GAL6-knockdown cells, compared with those in shCTL control cells. Interestingly, ST3GAL6 depletion by siRNAs could reduce both cell invasion and cell migration using transwell invasion and wound healing assays, respectively (*p* < 0.001; Figure 5F-I). Consistently, ST3GAL6 knockdown by shRNAs could also reduce the cell invasion capacity by transwell invasion assay (*p* < 0.001) (Figure S7B-C).

### ST3GAL6 reversed GATA3's effects on global sialylation level and cell invasion

**Figure 6:**
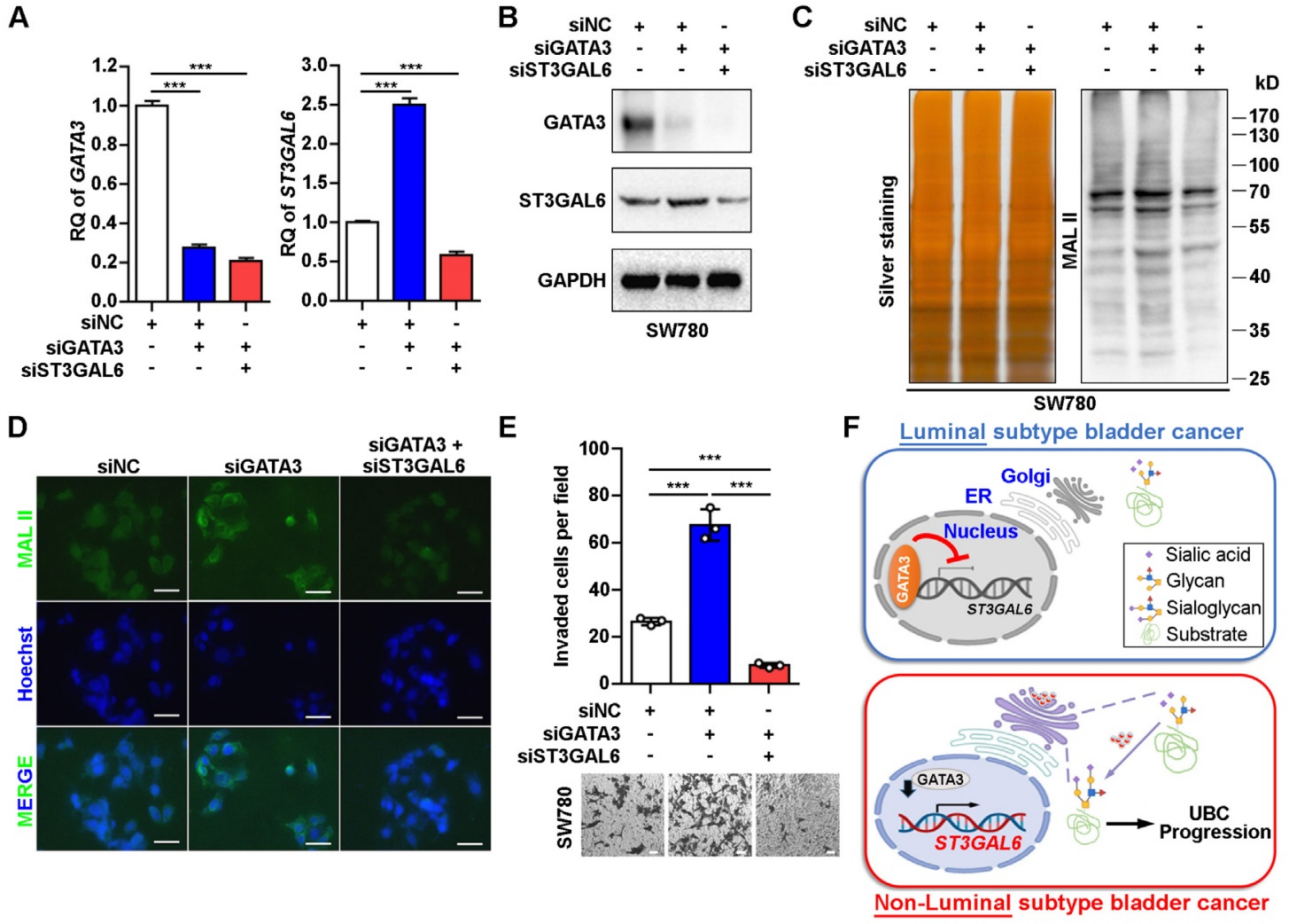
** ST3GAL6 reversed the increase of UBC cell invasion driven by GATA3 depletion. A**-**B**, mRNA and protein expression levels of GATA3 and ST3GAL6 in GATA3 siRNA alone or combined with ST3GAL6 siRNA treated SW780 cells, detected by qRT-PCR (**A**) and Western blotting (**B**), respectively. **C**-**D**, Global sialylation levels in SW780 cells transfected with GATA3 siRNA (siGATA3) alone or combined with ST3GAL6 siRNA (siST3GAL6), detected by MAL II blotting (**C**, right panel) and immunofluorescence staining (**D**, upper panel), respectively. The silver staining blot (**C**, left panel) was used as a loading control. Hoechst 33253 was used for the nuclei staining (**D**, middle panel). Scale bars, 50 µm.** E**, Transwell assay showed the cell invasion capacities of SW780 cells transfected with GATA3 siRNA alone or combined with ST3GAL6 siRNA. Scale bars, 50 µm. **F**, The working model of GATA3/ST3GAL6 axis in different subtypes of UBC, which is associated with glycogene expression and patients' outcomes. Data were presented as mean ± SD of three independent experiments; ***, p < 0.001.

In order to examine whether ST3GAL6 plays a key role in luminal specific marker GATA3-associated cell phenotype, we knocked down GATA3 alone, or both GATA3 and ST3GAL6 in the luminal-type SW780 UBC cells (Figure 6A and B). The increased α-2,3 sialylation by GATA3 depletion was reversed with the knockdown of ST3GAL6 gene, which were detected by MAL II blotting (Figure 6C) and immunofluorescence staining (Figure 6D). In addition, we observed that the loss of GATA3 increased SW780 cell invasiveness; however, co-depletion of ST3GAL6 and GATA3 significantly reduced cell invasion compared to GATA3 deletion alone (Figure 6E). Different roles of the glycogene ST3GAL6 in different subtypes of UBC patients for tumor progression were indicated (Figure 6F).

## Discussion

Post-translational modifications including glycosylation play key roles in UBC development. In this study, to our knowledge, we are the first to provide a global and unbiased approach to identify a novel 14-glycogene signature for glycogene-type based classification and prediction of clinical outcomes in UBC patients by integrating the transcriptomic data and corresponding survival information. This glycogene-type based classification was validated in a total number of 962 UBC patients derived from four independent datasets. From this 14-glycogene signature, overexpressed ST3GAL6 was further identified to be positively associated with tumor aggressiveness and poor prognosis in UBC patients, from both public datasets and our own cohorts.

Multiple strategies for molecular classification of UBCs have been reported 5, 6, 8-10, 37. Molecular subtypes, including luminal, basal, squamous-differentiation, epithelial-mesenchymal, cancer-stem cell, Claudin-low, p53-like, and neuroendocrine, started to be used for predictions of clinical outcomes and therapeutic interventions. However, most of these classifications were based on complicated gene expression patterns; the underlying molecular mechanisms are still to be investigated. Herein, we simplified the expression pattern to a 14-gene signature, and furthermore a glycogene (ST3GAL6), whose expression level was negatively correlated with luminal feature, as well as the positive associations with other features (including basal feature) in the majority of 12 datasets (n=2,130 in total). The novel finding that luminal-specific transcriptional factor GATA3 suppressed ST3GAL6 gene transactivation provided a possible mechanistic evidence for the negative association between ST3GAL6 mRNA level and the subtype with luminal feature in UBCs.

Aberrant global glycosylation has been implicated in cancer development and associated with cell adhesion, invasion and metastasis. The elevated levels of sLe^A^ and sLe^x^, which are essential for the function of selectin ligands in the adhesion of cancer cells onto the endothelium during metastasis, are frequently utilized as serum biomarkers for pancreatic cancer 38. A quantitative and qualitative measure of glycosylation is currently dependent on sequential tandem mass spectrometry analysis coupled to liquid chromatography and ion mobility spectrometry 39. However, precise glycan structural characterization of often isomeric glycans is challenging to be implemented in biological samples due to glycan separation, complex glycan mixtures, reduced resolution, and even cost 40. Because the type and amount of glycosylation of cancer cell mainly rely on the activity of glycosyltransferases and glycosidases 20, 41, we used an alternative strategy focusing on the expression levels of glycogenes from the numerous public transcriptomic data, and identified the glycogene, ST3GAL6, as a potential biomarker for molecular stratification in UBCs.

ST3GAL6 is one member of the sialyltransferase subfamily, called as ST3Gal (α2,3-ST), which functions to transfer sialic acid to 3'-hydroxyl group of a Gal residue 42. The overexpression of ST3GAL6 has been reported in multiple cancers, such as breast cancer, multiple myeloma and hepatocellular carcinoma, whereas low expression level in their normal counterparts 43-45. Dysregulation of ST3GAL6 promoted hepatocellular carcinoma and colon cancer cell proliferation and invasion via PI3K/AKT signaling, enhanced homing and survival of multiple myeloma in bone marrow niche 45, 46. Forced expression of ST3GAL6 also increased the resistance of gastric cancer cells to a Met tyrosine kinase receptor inhibitor crizotinib, with the compensatory activation of insulin receptor 47. In this study, we showed that ST3GAL6 expression was required for UBC cell invasion, but not proliferation. Though only a few receptors or ligands on tumor cell surface have been suggested as candidates for ST3GAL6, until recently a study indicated that ST3GAL6 was required for the α2,3-sialylation of EGFR, but not that of integrin β1 in HeLa cells by knockout approach 35. On the contrary, the downregulation of ST3GAL6 was observed in hepatocellular carcinoma, and was not associated with the levels of CD75s- and iso-CD75s-ganglioside content 48. The complicated role of ST3GAL6 in carcinogenesis is suggested; to explain such discrepancy, the accurate quantitative analysis method, precise application to clinical diagnosis and well characterization of the function and putative substrates of ST3GAL6 in each cancer type should be explored.

ST3GAL6 is reported to be regulated at multiple levels. Under hypoxic or inflammatory conditions, stabilized HIF1α and IL6 or IL8 induced ST3GAL6 at transcriptional level in MDA-MB-231 and human bronchial mucosa cells, respectively 49. LncRNA ST3GAL6-AS1, overlapping with ST3GAL6 at genomic level, positively regulated its host gene ST3GAL6 by recruiting MLL1 protein to enhance H3K4me3 level at ST3GAL6 promoter region 46. In addition, ST3GAL6 could also be regulated by non-coding RNAs. In liver cancer, miR-26a repressed ST3GAL6 at post-transcriptional level through binding to its 3'UTR 45. Herein, we added a new piece of evidence, showing that ST3GAL6 was down-regulated by the transcription factor GATA3, which is one of the key factor for the maintenance of luminal differentiation of urothelial cells 31. In non-luminal UBC subtypes, the downregulation of GATA3 may release its repression on ST3GAL6 promoter activity, induce ST3GAL6 expression and thus increase the global sialylation level in UBC cells for more malignant outcomes, such as increased capability for cell migration and invasion (Figure 6F). It will be intriguing to identify the substrates of ST3GAL6 in UBC cells, which may unveil the molecular mechanism of poor prognosis in ST3GAL6-active UBC subtype.

## Conclusions

In this study, we successfully identified a novel 14-gene signature for glycogene-type classification of UBC patients. ST3GAL6 gene, from this signature, was demonstrated to be regulated negatively by a luminal-specific transcriptional factor GATA3 and involved in UBC cell migration and invasion. ST3GAL6 as a potential biomarker for prediction of poor outcomes was also suggested in UBC patients.

## Supplementary Material

Supplementary figures and tables.

## Abbreviations

UBC: Urinary bladder cancer
TNM: Tumor-Node-Metastasis
NMIBC: non-muscle-invasive bladder cancer
MIBC: muscle-invasive bladder cancer
PUNLMP: papillary urothelial neoplasm of low malignant potential
EMT: epithelial to mesenchymal transition
sLe^A^: sialyl Lewis A
sLe^X^: sialyl Lewis X
ST3GAL6: ST3 beta-galactoside alpha-2,3-sialyltransferase 6
TCGA: The Cancer Genome Atlas
GEO: Gene Expression Omnibus
GGDB: glycogene database
NMF: non-negative matrix factorization
GSVA: gene set variation analysis
IHC: immunohistochemistry
HRP: horseradish peroxidase
NDP: nanozoomer digital pathology
qRT-PCR: quantitative reverse transcription polymerase chain reaction
MAL II: Maackia amurensis Lectin II
WT: wild-type
MT: mutant
IVIS: *in vivo* imaging system
CCK-8: Cell Counting Kit-8
PBS: Phosphate Buffered Saline
OS: overall survival
DFS: disease free survival
SD: standard deviation
