# Supplementary material for: Identification of glycogene-type and validation of ST3GAL6 as a biomarker predicts clinical outcome and cancer cell invasion in urinary bladder cancer
Source: Theranostics. 2020 Aug 8;10(22):10078–91. doi: 10.7150/thno.48711 (PMC7481430; doi:10.7150/thno.48711)
Supplement: Supplementary file 1 — Supplementary figures and tables. [file thnov10p10078s1.pdf]

Supplementary Information

Identification of glycogene-type and validation of ST3GAL6 as a biomarker predicts clinical outcome and cancer cell invasion in urinary bladder cancer

Sumiya Dalangood[1,2*], Zhen Zhu[1*], Zhihui Ma[2,3], Jiaxuan Li[1], Qinghe Zeng[2,3], Yilin Yan[4], Bing Shen[4], Jun Yan[5,6#], Ruimin Huang[2,3#]

1. MOE Key Laboratory of Model Animals for Disease Study, Model Animal Research Center of Nanjing University, Nanjing 210061, China
2. State Key Laboratory of Drug Research, Shanghai Institute of Materia Medica, Chinese Academy of Sciences, Shanghai 201203, China
3. University of Chinese Academy of Sciences, Beijing 100049, China
4. Department of Urology, Shanghai General Hospital, Shanghai Jiaotong University School of Medicine, Shanghai 200080, China
5. Department of Laboratory Animal Science, Fudan University, Shanghai 200032, China
6. Model Animal Research Center of Nanjing University, Nanjing 210061, China

*These authors contributed equally to this article.

#Corresponding authors:

Jun Yan, Ph.D., Department of Laboratory Animal Science, Fudan University, Shanghai 200032, China; Phone: 86-21-54237454; Email: yan_jun@fudan.edu.cn;

Ruimin Huang, Ph.D., Shanghai Institute of Materia Medica, Chinese Academy of Sciences, Shanghai 201203, China; Phone: 86-21-50817066; Email: rmhuang@simm.ac.cn

Contents

1. **Supplementary Figure S1.** Overview for the analysis procedures in this study …… 3
2. **Supplementary Figure S2.** A 14-gene signature for UBC glycogene-type classification …………………………………………………………………………….....4
3. **Supplementary Figure S3.** The correlations between mRNA expressional levels of 14-glycogene signature and 6 molecular features in UBC patients ……………..…..5
4. **Supplementary Figure S4.** The correlations between mRNA expression level of ST3GAL6 and clinical features in UBC patients ……....…………………….....…....6
5. **Supplementary Figure S5.** The Pearson correlation of mRNA expression levels between ST3GAL6 and GATA3 gene in five independent UBC cohorts ……………7
6. **Supplementary Figure S6.** The conserved GATA3 binding site in the intron 1 of ST3GAL6 is required for GATA3 to downregulate ST3GAL6 expression ………….8
7. **Supplementary Figure S7.** Biological effects of ST3GAL6 knockdown by shRNAs on UBC cells ..……………………………………………………………………..…….9
8. **Supplementary Table S1.** List of oligonucleotide sequences and reagents ….………10
9. **Supplementary Table S2.** The clinicopathological associations among 4 clusters based on glycogene-type in TCGA-BLCA provisional dataset ……………………….…….12
10. **Supplementary Table S3.** The 14 glycogenes differentiated the subcluster B1 from other three subclusters in TCGA provisional dataset …….…………….….……….13
11. **Supplementary Table S4.** Univariate and multivariate analyses of 14-glycogene signature for patients' outcomes in TCGA provisional dataset …………………...14
12. **Supplementary Table S5.** Univariate and multivariate analyses of 14-glycogene signature for overall survival in GSE32894 dataset …………………..………..15
13. **Supplementary Table S6.** Univariate and multivariate analyses of 14-glycogene signature for patients' outcomes in GSE13507 dataset ……………….….……..16
14. **Supplementary Table S7.** The association between the protein expression level of ST3GAL6 and clinicopathological parameters in 52 UBC samples from our IHC cohort …………………………………………………………………...………...17
15. **Supplementary Table S8.** Univariate analysis of ST3GAL6 expression with overall survival in 52 UBC samples from our IHC cohort ………..………………….……...... 18

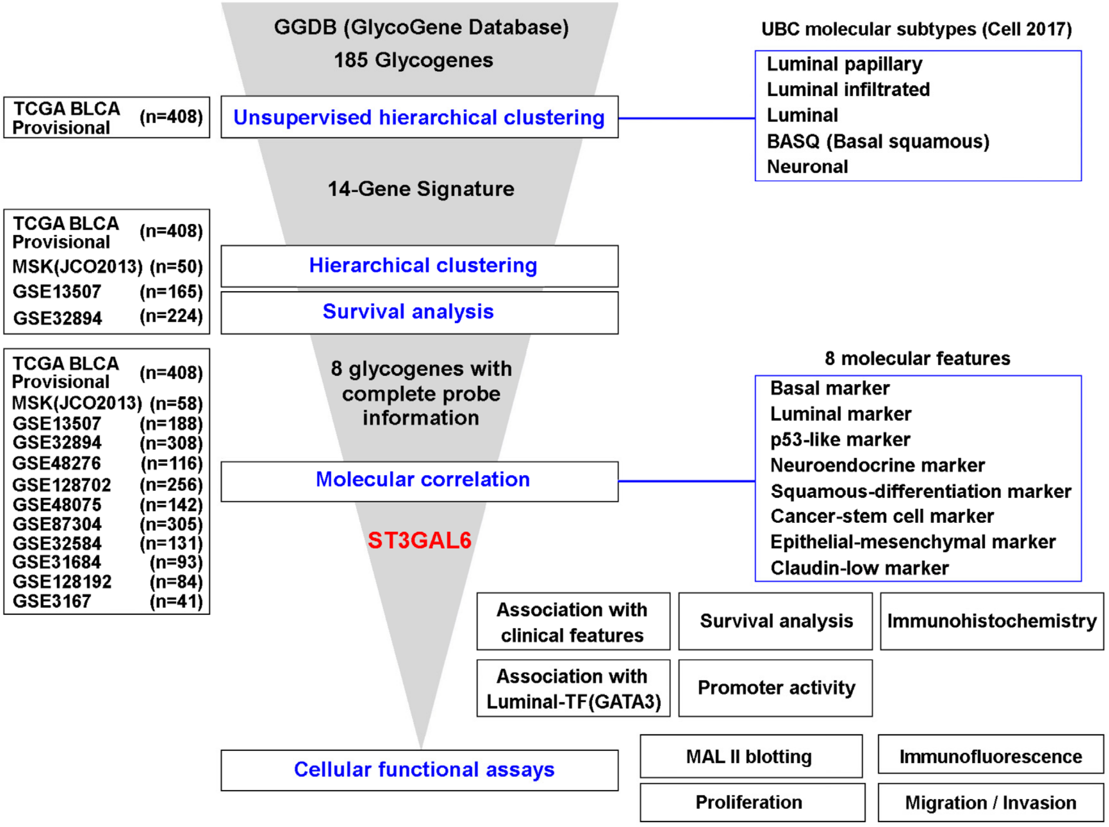

Supplementary Figure S1. Overview for the analysis procedure in this study. 1) Unsupervised hierarchical clustering of 185 glycogenes in TCGA-BLCA provisional dataset, showing the high correlation between glycogene-types and molecular subtypes of UBC (Cell 2017). 2) Using hierarchical clustering along with survival analysis, a 14-glycogene signature for glycogene-type classification was identified and confirmed in TCGA-BLCA provisional, MSK (JCO 2013), GSE13507, and GSE32894 datasets. 3) By Pearson correlation analysis, molecular correlation between 8 glycogenes from the 14-glycogene signature with complete probe information and 8 molecular features for UBC patients was analyzed. In 12 datasets, ST3GAL6 gene was shown consistent changes, negative association with Luminal subtype. 4) Correlations between ST3GAL6 gene expression and tumor stage, grade, lymph node invasion, recurrence, survival, and GATA3 gene expression were analyzed. 5) Bioinformatics prediction for transcriptional factor binding site, promoter activity, MAL II blotting, immunofluorescence staining, cell proliferation, cell migration (wound-healing) and cell invasion (transwell) assays were performed to investigate the negative regulation of GATA3, luminal-specific transcription factor, on ST3GAL6 gene expression and further functions.

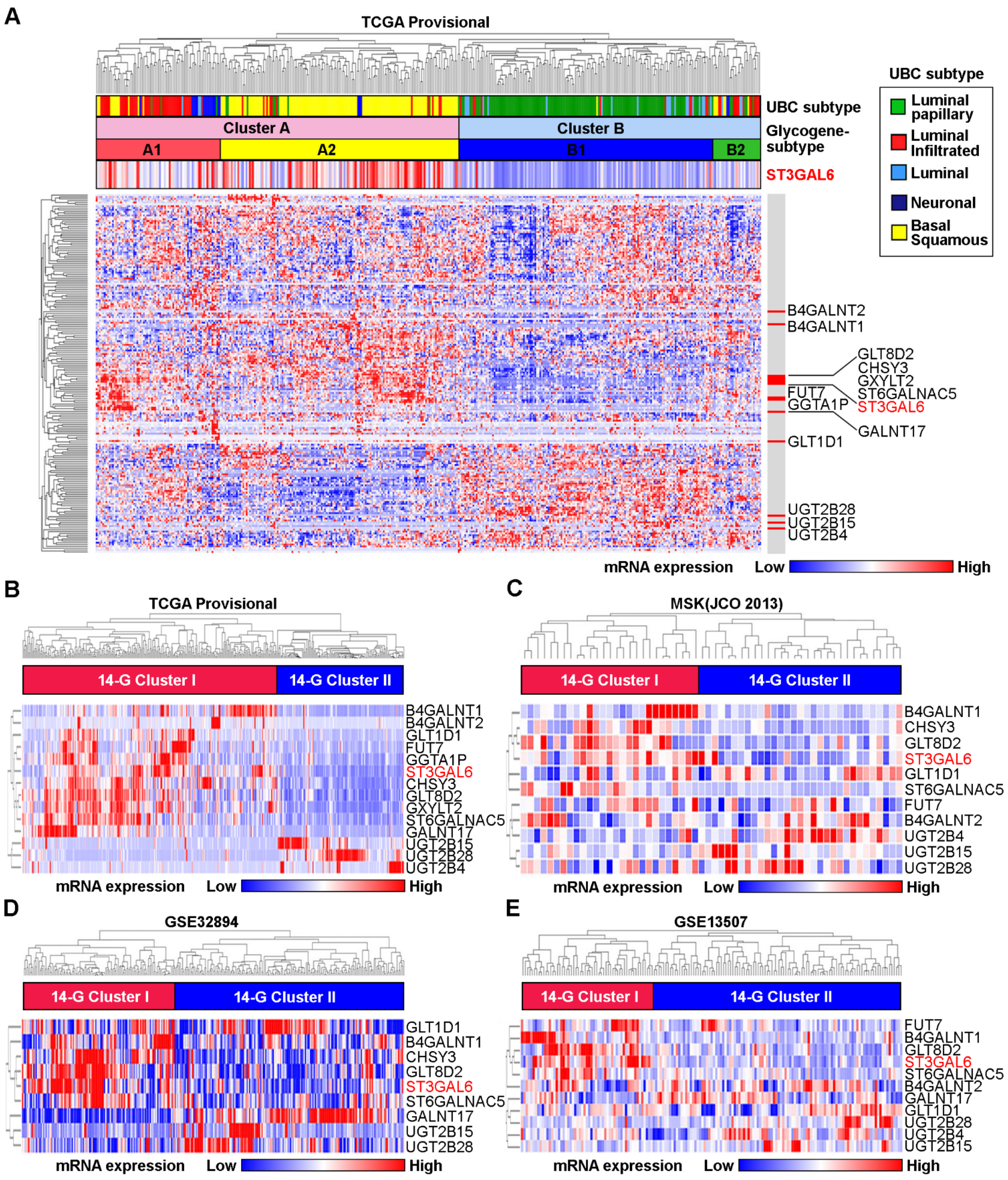

Supplementary Figure S2. A 14-gene signature for UBC glycogene-type classification. A, 14 glycogenes were identified to be significantly dysregulated in subcluster B1 comparing with subclusters A1, A2 and B2 (TCGA provisional dataset). This 14-glycogene signature allowed the glycogene-based classification (14-G Cluster I and II) in UBC patients from TCGA provisional (**B**), MSK (JCO 2013, **C**), GSE32894 (**D**) and GSE13507 (**E**) datasets.

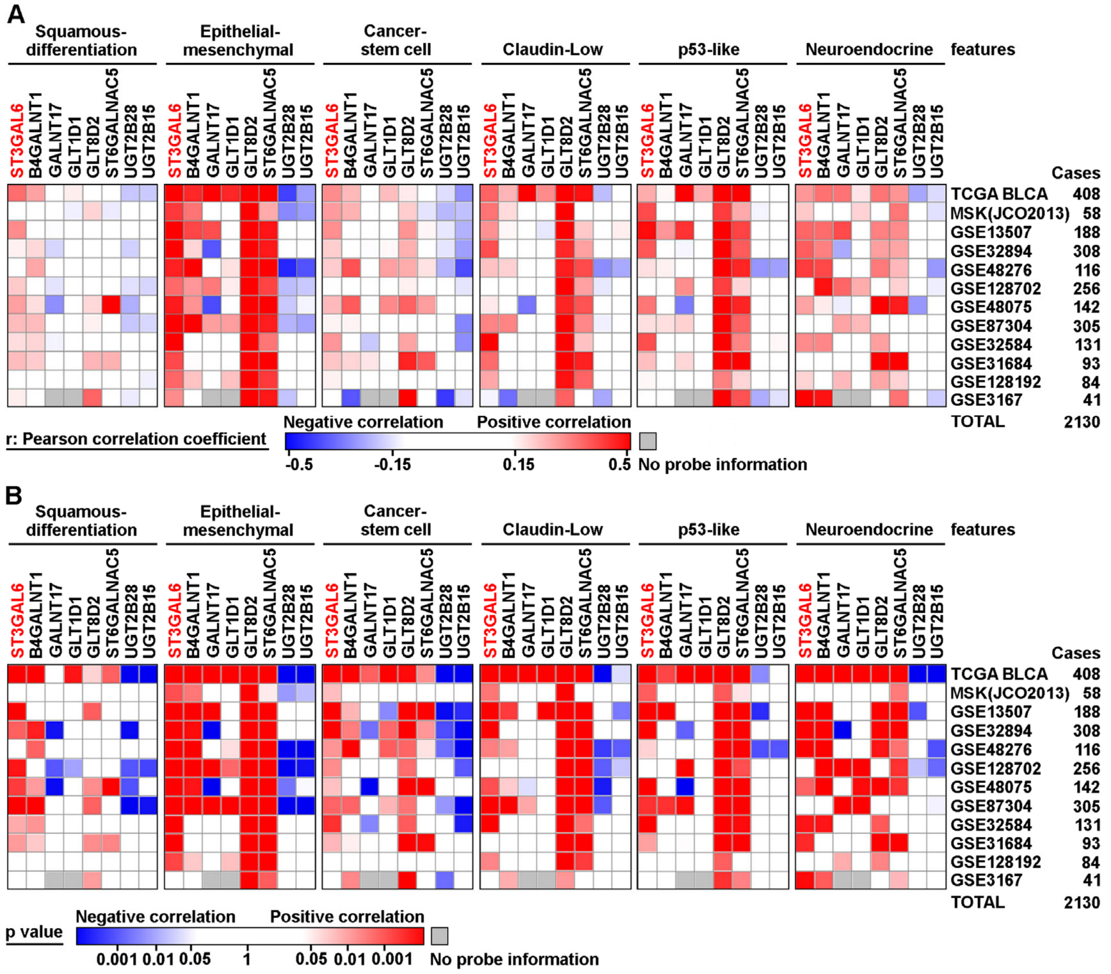

Supplementary Figure S3. The correlations between mRNA expressional levels of 14-glycogene signature and 6 molecular features in UBC patients. Pearson correlation analyses (r value, **A**) with the statistical significance of correlation (p value, **B**) were performed between the expression levels of 8 glycogenes from the 14-gene signature with the complete probe information and 6 molecular features (Squamous-differentiation, Epithelial-mesenchymal, cancer-stem cell, Claudin-low, p53-like and neuroendocrine) in 12 independent cohorts (n=2,130 in total). Colored-blocks in the heatmaps represented the glycogenes with upregulation (in red) or downregulation (in blue), respectively.

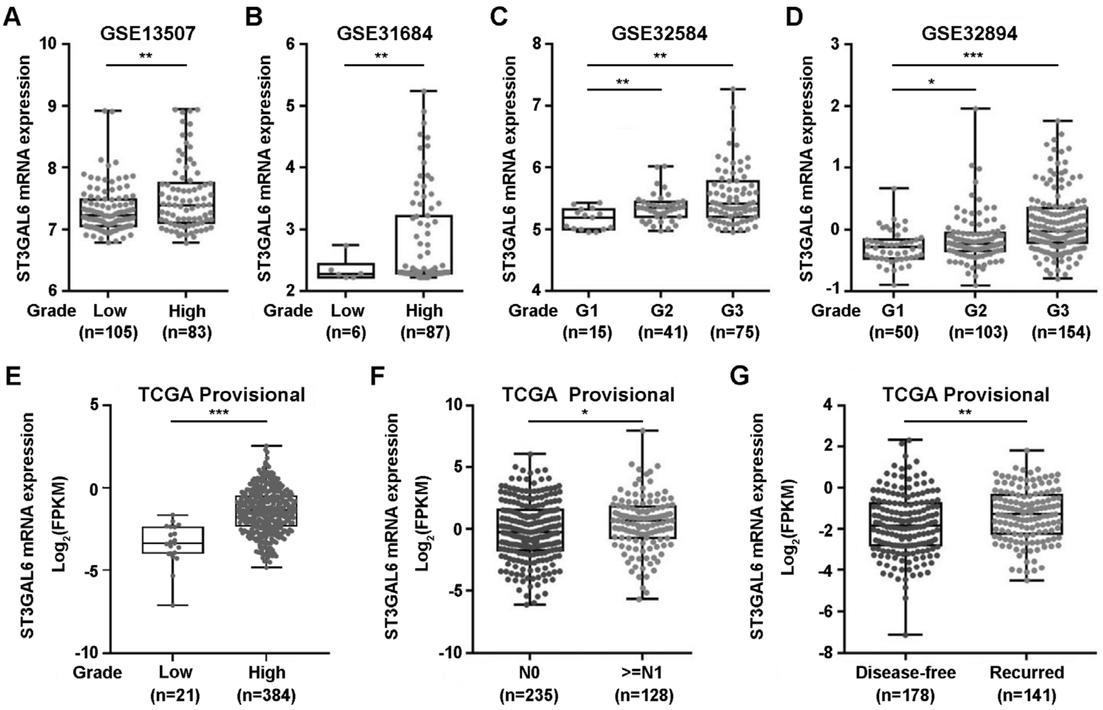

Supplementary Figure S4. The correlations between mRNA expression level of ST3GAL6 and clinical features in UBC patients. A-E, The correlation between ST3GAL6 mRNA levels and tumor grade of UBC patients from GES13507 (**A**), GES31684 (**B**), GES32584 (**C**), GES32894 (**D**), and TCGA provisional (**E**) datasets. The association of ST3GAL6 mRNA levels with the status of lymph node metastasis (**F**) and recurrence (**G**) in TCGA provisional dataset. G1, grade 1; G2, grade 2; G3, grade 3. >=N1, with lymph node metastasis; N0, without lymph node metastasis. *, $p < 0.05$; **, $p < 0.01$; ***, $p < 0.001$.

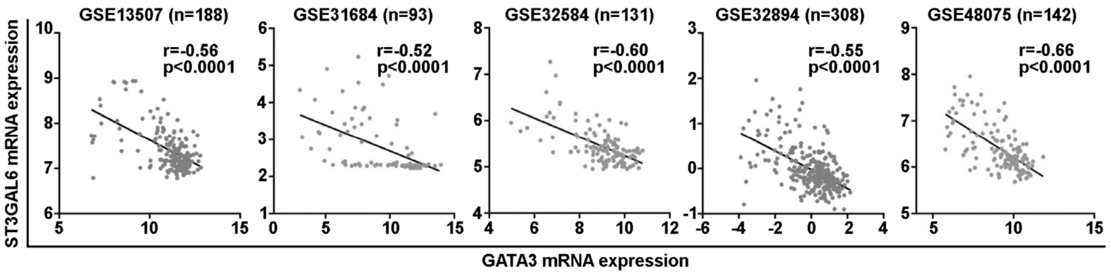

Supplementary Figure S5. The Pearson correlation of mRNA expression levels between ST3GAL6 and GATA3 genes in five independent UBC cohorts.

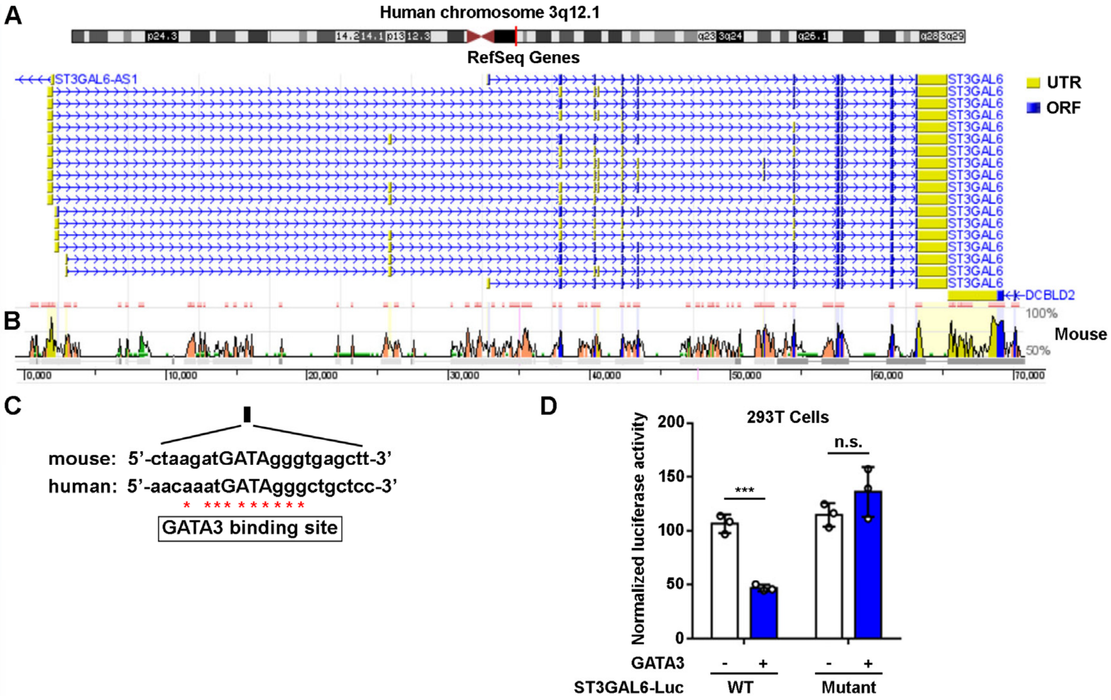

Supplementary Figure S6. The conserved GATA3 binding site in the intron 1 of ST3GAL6 is required for GATA3 to downregulate ST3GAL6 expression. A, The genomic structure of *ST3GAL6* gene on human chromosome 3q12.1. Yellow box, untranslated region (UTR). Blue box, open reading frame (ORF). **B,** The plot of conserved rate between human and mouse *ST3GAL6* gene, which was analyzed on Comparative Genomics Developments website (www.dcode.org). **C,** The location and comparison of conserved GATA3 binding sites between human and mouse *ST3GAL6* loci. **D,** The promoter activity from ST3GAL6 luciferase reporters (ST3GAL6-Luc), driven by the ST3GAL6 promoter fragment containing the wildtype (WT) or mutant GATA3 binding site. GATA3 expression plasmid were co-transfected with ST3GAL6 luciferase reporter and pRL-CMV plasmid into 293T cells. pCDH-3XFLAG was used as control plasmid for GATA3 expression plasmid. ***, $p < 0.001$; n.s., $p \geq 0.05$.

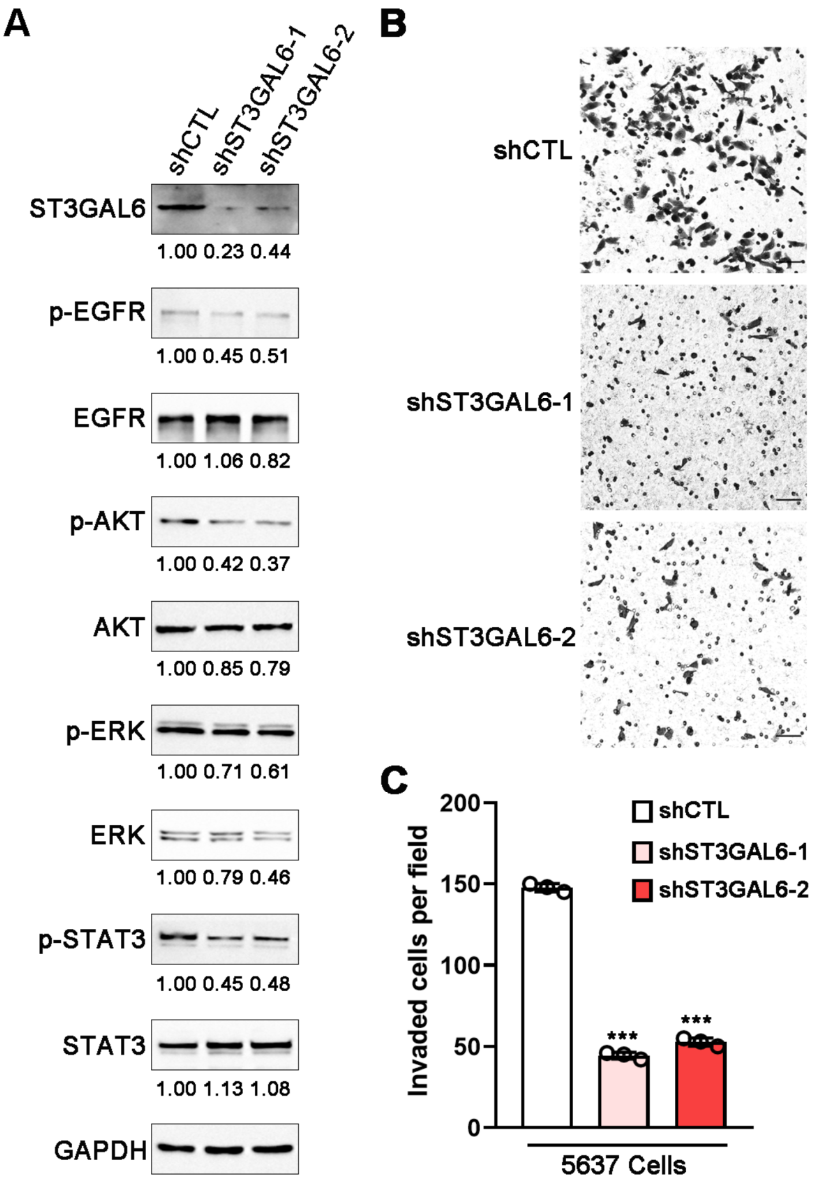

Supplementary Figure 7. Biological effects of ST3GAL6 knockdown by shRNAs on UBC cells. A, Effects of ST3GAL6 knockdown by shRNAs on RAS-RAF-MEK-ERK, PI3K-AKT and JAK-STAT pathways in 5637 UBC cells, detected by Western blotting analysis. The normalized protein level (the ratio of test protein to GAPDH protein from same sample) was indicated below each band by ImageJ software. **B,** Effects of ST3GAL6 knockdown by shRNAs on cell invasion in 5637 UBC cells, detected by transwell invasion assay. Scale bars, 50 μm. **C,** Quantifications of images from the transwell invasion assay. Data were presented as mean ± SD of three independent experiments; ***, $p < 0.001$.

Supplementary Table S1. List of oligonucleotide sequences and reagents

	Direction	Sequences (5'-3')
**qRT-PCR primers**		
GATA3	Forward	GCCCCTCATTAAGCCCAAG
	Reverse	TTGTGGTGGTCTGACAGTTCG
ST3GAL6	Forward	ATTGCCATCACATTGGCGTTT
	Reverse	GCAAAGGACTCTTGAGGTCAG
β-actin	Forward	CATGTACGTTGCTATCCAGGC
	Reverse	CTCCTTAATGTCACGCACGA
**PCR primers for promoter activity assays**		
pGL3-ST3GAL6 -promoter (WT)	Forward	TTTCTCTATCGATAGGTACCTTCAGAGGTCAGGGATAGAGC
	Reverse	CTTAGATCGCAGATCTCGAGCATTTCTCTTCTGGGGCAGAG
pGL3-ST3GAL6 -promoter (mutant GATA3 binding site)	Overlapping Forward	AACAAATGA**GC**GGGCTGCTCCATCTGCTTGGTCGGTTTTC
	Overlapping Reverse	GGAGCAGCCC**GC**TCATTTGTTCATCTATCCATATATCCATGTATCC
pCDH-3XFLAG -GATA3	Forward	ACAAGCTTGCGGCCGCGATGGAGGTGACGGCGGA
	Reverse	TGCCCTCGATATCGAATTCACCCATGGCGGTGACC
**siRNA and shRNA sequences (Sense 5'-3')**		
siGATA3	siGATA3-1	CUCAUUAAGCCCAAGCGAATT
	siGATA3-2	GGGCUCUACUACAAGCUUCTT
siST3GAL6	siST3GAL6-1	CAGCCUUAAACCUGAUUUATT
	siST3GAL6-2	CGACUCCUAUGAUGUAAUATT
siRNA control	siNC	UUCUCCGAACGUGUCACGUTT
shST3GAL6	shST3GAL6-1	CCGGTCCTCTATTATGTACTGCATTCTCGAGAATGCAGTACATAATAGAGGATTTTTG
	shST3GAL6-2	CCGGGATGAGAACATCAGCGGAATACTCGAGTATTCCGCTGATGTTCTCATCTTTTTG
shRNA control	shCTL	CCGGGCGCGATAGCGCTAATAATTTCTCGAGAAATTATTAGCGCTATCGCGCTTTTT

Antibody	Company	Cat #	RRID	Application (Dilution)
GATA3	BD	#558686	AB_2108590	WB (1: 500)
GAPDH	Santa Cruz	sc-47724	AB_627678	WB (1: 1,000)
MAL II	Vector Labs	B-1265	AB_2336569	WB (1:1,000); IF (1:100)
ST3GAL6	Abcam	Ab106527	AB_10865191	WB (1:500); IHC (1:100)
EGF Receptor	CST	#4267	AB_2246311	WB (1:1,000)
phospho-EGF Receptor (Tyr1068)	CST	#3777	AB_2096270	WB (1:1,000)
AKT (pan)	CST	#4691	AB_915783	WB (1:1,000)
phospho-AKT (Ser473)	CST	#4060	AB_2315049	WB (1:1,000)
p44/42 MAPK (ERK1/2)	CST	#4695	AB_390779	WB (1:1,000)

Cont'd				
phospho-p44/42 MAPK (ERK1/2) (Thr202/Tyr204)	CST	#4370	AB_2315112	WB (1:1,000)
STAT3	CST	#9139	AB_331757	WB (1:1,000)
phospho-STAT3 (Tyr705)	CST	#9145	AB_2491009	WB (1:1,000)
HRP-conjugated streptavidin	Vector Labs	SA-5704	N.A.	WB (ready to use)
FITC-Avidin	BioLegend	#405101	N.A.	IF (1:1,000)
Goat α-rabbit HRP 2nd antibody	Jackson Immuno-Research	#846738	N.A.	WB (1:5,000)
Goat α-mouse HRP 2nd antibody		#847273	N.A.	WB (1:5,000)

Kit and Reagents	Company	Cat #
AceQ Universal SYBR qPCR Master Mix	Vazyme	Q511-02
Cell Counting Kit-8 (CCK-8)	Vazyme	A311-01
CloneExpress II One Step Cloning Kit	Vazyme	C112
Corning BioCoat 24-Multiwell Tumor Cell Invasion Systems	Corning	#354165
DAB visualization kit	Maixin_Bio	DAB-0031
Dual Luciferase Reporter Gene Kit	Beyotime	RG027
EDTA-free Protease Inhibitor Cocktail	Roche	#4693132001
Fast Silver Stain Kit	Beyotime	P0017S
Hoechst	Thermo Scientific	#33342
Lipofectamine 3000	Invitrogen	L3000008
Lipofectamine RNAiMAX reagent	Invitrogen	#13778
Mut Express II Fast Mutagenesis Kit V2	Vazyme	C214-02
QuantiTect Reverse Transcription Kit	QIAGEN	#205311
SuperSignal West Pico PLUS Chemiluminescent Substrate	Thermo Scientific	#34579
Trizol reagent	Invitrogen	#15596026

Supplementary Table S2. The clinicopathological associations among 4 clusters based on glycogene-type in TCGA-BLCA provisional dataset

	Total (n=408)	Cluster A1 (n=75)	Cluster A2 (n=146)	Cluster B1 (n=154)	Cluster B2 (n=33)	Cluster B1 *vs* Others p
**Age (years)**						0.1361
**Mean±SD**	68.07 ± 10.61	68.65 ± 8.88	67.93 ± 10.67	67.08 ± 11.54	72.15 ± 8.57	
**Gender**						0.1039
**Male**	301	59	98	121	23	
**Female**	107	16	48	33	10	
**Grade**						**< 0.0001**
**Low**	21	0	1	20	0	
**High**	384	74	145	132	33	
**NA**	3	1	0	2	0	
**Stage**						**< 0.0001**
**I**	2	1	0	1	0	
**II**	130	17	32	70	11	
**III**	140	27	62	43	8	
**IV**	134	30	52	38	14	
**NA**	2	0	0	2	0	

Numbers in bold indicate p value with statistical difference.

Supplementary Table S3. The 14 glycogenes differentiated the subcluster B1 from other three subclusters in TCGA provisional dataset

Gene Symbol	GENE ID	Description	Cluster B1 *vs* Others p
**B4GALNT1**	2583	beta-1,4-N-acetyl-galactosaminyl-transferase 1	2.68E-28
**B4GALNT2**	124872	beta-1,4-N-acetyl-galactosaminyl-transferase 2	9.76E-07
**CHSY3**	337876	chondroitin sulfate synthase 3	1.66E-34
**FUT7**	2529	fucosyltransferase 7	1.25E-18
**GALNT17**	64409	Polypeptide N-acetylgalactosaminyltransferase 17	4.96E-19
**GGTA1P**	2681	glycoprotein, alpha-galactosyltransferase 1 pseudogene	4.73E-23
**GLT1D1**	144423	glycosyltransferase 1 domain containing 1	9.31E-30
**GLT8D2**	83468	glycosyltransferase 8 domain containing 2	6.30E-33
**GXYLT2**	727936	glucoside xylosyltransferase 2	1.65E-43
**ST3GAL6**	10402	ST3 beta-galactoside alpha-2,3 -sialyltransferase 6	2.38E-44
**ST6GALNAC5**	81849	ST6 N-acetylgalactosaminide alpha-2,6 -sialyltransferase 5	2.15E-17
**UGT2B15**	7366	UDP glucuronosyltransferase family 2 member B15	4.01E-12
**UGT2B28**	54490	UDP glucuronosyltransferase family 2 member B28	1.39E-14
**UGT2B4**	7363	UDP glucuronosyltransferase family 2 member B4	6.45E-03

Supplementary Table S4. Univariate and multivariate analyses of 14-glycogene signature for patients' outcomes in TCGA provisional dataset

Variables	Univariate analysis		Multivariate analysis	
	HR (95% CI)	p	HR (95% CI)	p
**Overall Survival (OS)**				
Cluster (Cluster I vs Cluster II)	1.982(1.380-2.849)	**0.0002**	1.663(1.145-2.416)	**0.0076**
Age (Continuous)	1.032(1.017-1.048)	**<0.0001**	1.031(1.015-1.047)	**<0.0001**
Gender (Female vs Male)	0.882(0.639-1.217)	0.445	0.913(0.659-1.263)	0.581
Grade (Low vs High)	2.958(0.732-11.951)	0.128	1.281(0.307-5.343)	0.734
Stage (Stage I-III vs IV)	2.282(1.703-3.057)	**<0.0001**	2.057(1.528-2.769)	**<0.0001**
**Disease free survival (DFS)**				
Cluster (Cluster I vs Cluster II)	1.718(1.183-2.494)	**0.004**	1.385(0.944-2.030)	0.096
Age (Continuous)	1.014(0.998-1.030)	0.084	1.010(0.994-1.027)	0.224
Gender (Female vs Male)	0.905(0.630-1.300)	0.589	0.868(0.598-1.259)	0.455
Grade (Low vs High)	3.076(0.759-12.47)	0.116	1.802(0.428-7.585)	0.422
Stage (Stage I-III vs IV)	2.444(1.760-3.395)	**<0.0001**	2.282(1.624-3.205)	**<0.0001**

Abbreviations: HR, Hazard ratio; CI, Confidence interval.

Numbers in bold indicate p value with statistical difference.

Supplementary Table S5. Univariate and multivariate analyses of 14-glycogene signature for overall survival in GSE32894 dataset

Variables	Univariate analysis		Multivariate analysis	
	HR (95% CI)	p	HR (95% CI)	p
Cluster (Cluster I vs II)	2.500(1.133-5.513)	**0.023**	0.776(0.295-2.041)	0.608
Age (Continuous)	0.975(0.945-1.007)	0.125	1.003(0.942-1.068)	0.921
Gender (Female vs Male)	1.538(0.577-4.100)	0.389	1.387(0.446-4.310)	0.572
Grade (G1/G2 vs G3/G4)	10.173(3.0333-4.126)	**<0.0001**	0.428(0.076-2.423)	0.337
Stage (Ta/T1 vs T2-4)	45.087(10.617-191.473)	**<0.0001**	92.056(11.082-764.678)	**<0.0001**

Abbreviations: HR, Hazard ratio; CI, Confidence interval.

Numbers in bold indicate p value with statistical difference.

Supplementary Table S6. Univariate and multivariate analyses of 14-glycogene signature for patients' outcomes in GSE13507 dataset

Variables	Univariate analysis		Multivariate analysis	
	HR (95% CI)	p	HR (95% CI)	p
**Overall survival (OS)**				
Cluster (Cluster I vs II)	1.585(0.971-2.587)	0.065	1.525(0.895-2.598)	0.12
Age (Continuous)	1.070(1.044-1.096)	**<0.0001**	1.077(1.048-1.106)	**<0.0001**
Gender (Female vs Male)	0.641(0.361-1.138)	0.129	0.827(0.449-1.520)	0.54
Grade (Low vs High)	2.740(1.694-4.433)	**<0.0001**	1.171(0.650-2.107)	0.599
Stage (Ta/T1 vs T2-4)	2.730(1.688-4.415)	**<0.0001**	2.491(1.392-4.455)	**0.002**
**Cancer-specific survival (CSS)**				
Cluster (Cluster I vs II)	3.447(1.699-6.993)	**0.001**	2.547(1.107-5.861)	**0.028**
Age (Continuous)	1.051(1.016-1.088)	**0.004**	1.074(1.027-1.123)	**0.002**
Gender (Female vs Male)	0.477(0.22-1.032)	0.06	0.515(0.230-1.152)	0.106
Grade (Low vs High)	5.985(2.757-12.992)	**<0.0001**	1.114(0.472-2.629)	0.806
Stage (Ta/T1 vs T2-4)	17.897(6.251-51.244)	**<0.0001**	14.556(4.57-46.361)	**<0.0001**

Abbreviations: HR, Hazard ratio; CI, Confidence interval.

Numbers in bold indicate p value with statistical difference.

Supplementary Table S7. The association between the protein expression level of ST3GAL6 and clinicopathological parameters in 52 UBC samples from our IHC cohort

Characteristics	n	Expression of ST3GAL6 high（n, %）	Expression of ST3GAL6 low（n, %）	p
**Gender**				0.761
**Male**	40	22(55.0%)	18(45.0%)	
**Female**	12	6(50.0%)	6(50.0%)	
**Age**				0.634
**≥ 65**	30	17(56.7%)	13(43.3%)	
**< 65**	22	11(50.0%)	11(50.0%)	
**Tumor grade**				**0.029**
**Low**	24	9(37.5%)	15(62.5%)	
**High**	28	19(67.9%)	9(32.1%)	
**T stage**				0.238
**Ta-T1**	37	18(48.6%)	19(51.4%)	
**T2-T4**	15	10(66.7%)	5(33.3%)	
**N stage**				0.856
**N0**	47	25(53.2%)	22(46.8%)	
**≥ N1**	5	3(60.0%)	2(40.0%)	

Numbers in bold indicate p value with statistical difference.

Supplementary Table S8. Univariate analysis of ST3GAL6 expression with overall survival in 52 UBC samples from our IHC cohort

Variables	HR (95% CI)	p
Age (<60 vs ≥60)	2.325 (0.852-6.344)	0.099
Gender (Female vs Male)	1.327 (0.488-3.605)	0.579
T stage (Ta-T1 vs T2-T4)	2.435 (0.947-6.259)	0.065
Grade (Low vs High)	4.505 (1.650-12.299)	**0.003**
ST3GAL6 expression (Low vs High)	2.951 (1.149-7.579)	**0.025**

Abbreviations: HR, Hazard ratio; CI, Confidence interval.

Numbers in bold indicate p value with statistical difference.
